# Punched-out gluteal and perianal ulcerations

**DOI:** 10.1016/j.jdcr.2025.05.010

**Published:** 2025-06-10

**Authors:** Sarah Williamson, Julie Iacullo, Harry Winfield

**Affiliations:** Department of Dermatology, MetroHealth Medical Center, Cleveland, Ohio

**Keywords:** amyloid, amyloidosis, gluteal, multiple myeloma, perianal, ulcer

## Case presentation

**Fig 1:**
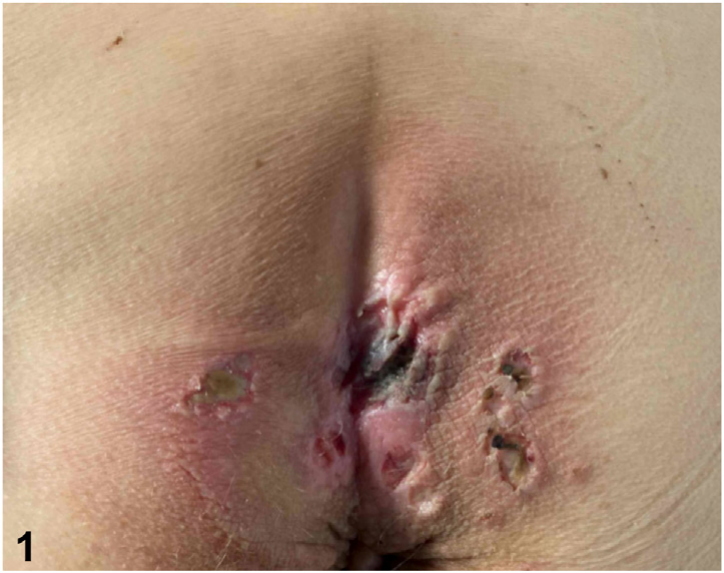

**Fig 2:**
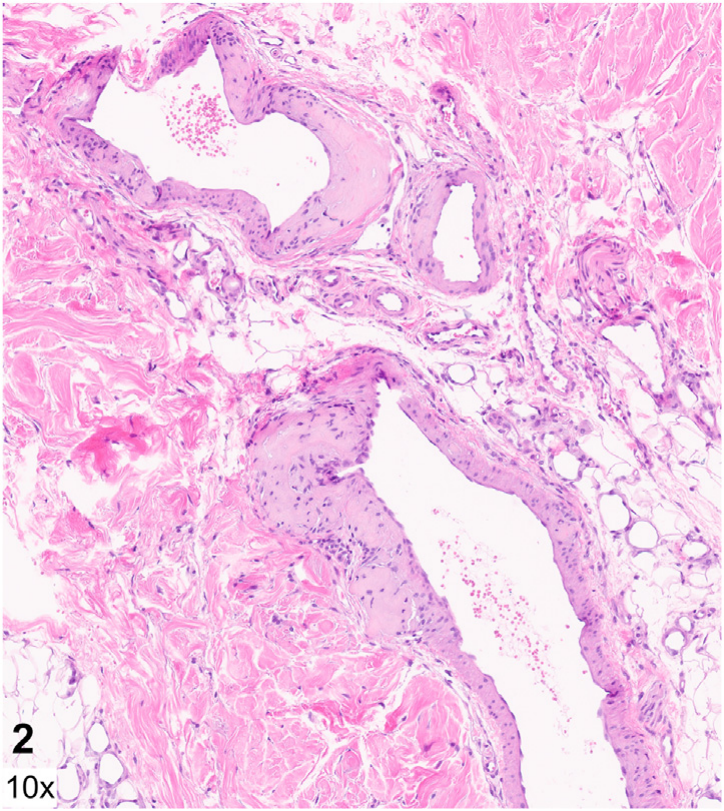

**Fig 3:**
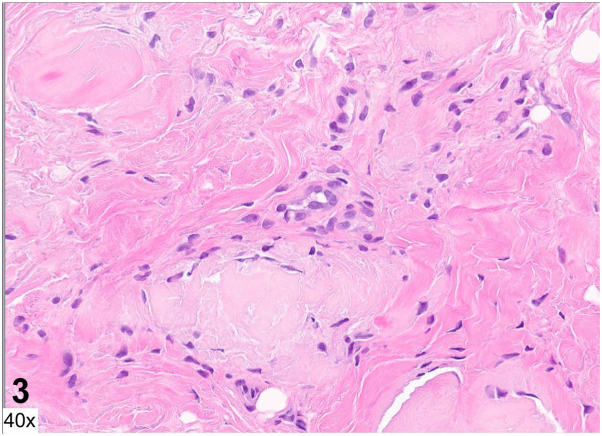

A 55-year-old man with no prior medical history was admitted for fatigue, lower back pain, and a 2-week history of well-demarcated painful ulcers within ill-defined red-brown plaques localized to the gluteal and perianal skin (Fig 1). He denied ulceration of other mucosal surfaces. He was found to have anemia, thrombocytopenia, renal failure, and hypercalcemia. Serum and urine protein electrophoresis identified a monoclonal lambda band, with a serum free lambda/kappa light chain ratio of 597.7 (reference range 0.26-1.65). Bone marrow biopsy revealed a plasma cell myeloma. Human immunodeficiency virus screening was negative. A punch biopsy was performed at the edge of a gluteal fold ulcer (Figs 2 and 3, hematoxylin and eosin; Fig 4, Congo red).

**Fig:**
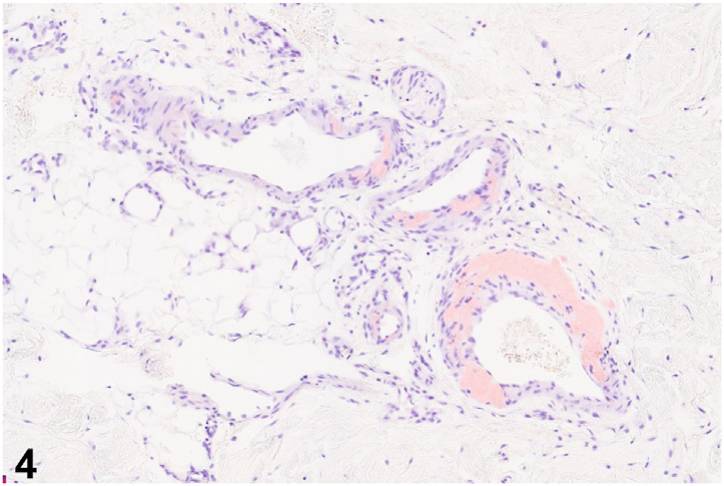
4

**Question 1: What is the most likely diagnosis?**

A. Behçet's disease
B. Cytomegalovirus infection
C. Amyloidosis
D. Herpes simplex virus infection
E. Crohn's disease

**Answers:**

A. Behçet's disease – Incorrect. Behçet's disease is a multisystem disorder characterized by recurrent genital and oral ulcers, as well as ophthalmologic disease including anterior uveitis, posterior uveitis, and retinal vasculitis. Oral ulcerations, which are a required diagnostic criterion, were absent in this case.
B. Cytomegalovirus infection – Incorrect. Patients with HIV can develop chronic perianal and gluteal ulcers secondary to cytomegalovirus infection. On histopathology, infected endothelial cells demonstrate an “owl's eye” appearance.
C. Amyloidosis – Correct. Histopathology revealed amyloid deposition within the dermis and dermal capillary and venule walls (Figs 2 and 3). Congo red staining highlights amyloid within venule walls (Fig 4). The results of the serum and urine protein electrophoresis, and elevated serum free lambda/kappa light chain ratio, were consistent with multiple myeloma.
D. Herpes simplex virus infection – Incorrect. Biopsy of the edge of an HSV ulcer would classically demonstrate an intraepidermal ulcer with ballooning keratinocyte degeneration, nuclear molding, chromatin margination, and multinucleated giant cells.
E. Crohn's disease – Incorrect. Cutaneous Crohn's disease presents with ulcers, knife-like fissures, plaques, sinus tracts, and perianal skin tags involving the gluteal skin. Noncaseating epithelioid granulomas in the dermis are characteristics on histopathology.

**Question 2: What is the correct classification for this patient's disease?**

A. Primary cutaneous lichen amyloidosis
B. Primary cutaneous anosacral amyloidosis
C. Secondary cutaneous amyloidosis
D. Primary systemic amyloidosis
E. Secondary systemic amyloidosis

**Answers:**

A. Primary cutaneous lichen amyloidosis – Incorrect. In lichen amyloidosis, amyloid is restricted to the upper dermis, especially the papillary dermis. Additionally, the overlying epidermis often has features of chronic rubbing, rather than ulceration. The amyloid is keratinocyte derived, in contrast to primary systemic amyloidosis. Lichen amyloidosis presents clinically as hyperpigmented papules coalescing into plaques, most often involving the extensor surfaces, including the shins and forearms.
B. Primary cutaneous anosacral amyloidosis – Incorrect. Anosacral amyloidosis has been reported as a rare variant of primary localized cutaneous amyloidosis, characterized by hyperkeratotic rippled brown plaques radiating from the anus to the buttocks.^1^ Ulcerations are not a well-established feature of this subtype of cutaneous amyloidosis. β2-microglobulin (hemodialysis-associated) amyloidosis is an additional variant of cutaneous amyloidosis has been reported to rarely involve the buttocks.^2^
C. Secondary cutaneous amyloidosis – Incorrect. Secondary cutaneous amyloidosis describes amyloid deposits observed incidentally within other skin tumors. These are clinically inapparent but can be detected on histopathology.
D. Primary systemic amyloidosis – Correct. Our patient's cutaneous and serologic findings, as well as his bone marrow biopsy results, were consistent with primary systemic amyloidosis secondary to multiple myeloma. Primary systemic amyloidosis occurs due to an underlying plasma cell dyscrasia, with deposition of AL protein composed of immunoglobulin light chains, usually lambda-type.
E. Secondary systemic amyloidosis – Incorrect. Secondary systemic amyloidosis occurs due to deposition of serum amyloid A protein, an acute phase reactant that is upregulated in chronic inflammatory diseases such as rheumatoid arthritis and tuberculosis. Secondary systemic amyloidosis rarely affects the skin and is more often noted in other organs such as the liver and kidney.

**Question 3: Which of the following statements is correct?**

A. Primary cutaneous anosacral amyloidosis has been described mostly in Caucasian populations
B. In bullous amyloidosis, amyloid deposits within the epidermis leading to an intraepidermal split
C. Primary cutaneous anosacral amyloidosis is characterized histologically by dermal and perivascular amyloid aggregates
D. Bullous amyloidosis is more often associated with primary cutaneous amyloidosis rather than primary systemic amyloidosis with multiorgan involvement
E. Congo red stain can highlight amyloid deposits around the sweat glands and within blood vessel walls in primary systemic amyloidosis

**Answers:**

A. Primary cutaneous anosacral amyloidosis has been described mostly in Caucasian populations – Incorrect. Primary cutaneous anosacral amyloidosis is a rare entity that has been reported primarily in Asian and Chinese-Caribbean patients to date.^3^
B. In bullous amyloidosis, amyloid deposits within the epidermis leading to an intraepidermal split – Incorrect. Bullous amyloidosis is characterized by amyloid deposits in the dermis causing a subepidermal or intradermal split.^3^^,^^4^ In our patient, we suspect his ulcerations occurred due to prolonged pressure on the sacrum while sitting or lying down, resulting in shearing of skin rendered fragile from dermal amyloid deposition.
C. Primary cutaneous anosacral amyloidosis is characterized histologically by dermal and perivascular amyloid aggregates – Incorrect. Primary cutaneous anosacral amyloidosis is characterized by papillary dermal amyloid deposition, hyperkeratosis, an increase in melanin in the basal layer, and pigment incontinence.^3^
D. Bullous amyloidosis is more often associated with primary cutaneous amyloidosis rather than primary systemic amyloidosis with multiorgan involvement – Incorrect. Bullous amyloid lesions have been described more commonly in association with primary systemic amyloidosis including myeloma-associated amyloidosis, though rare cares without systemic involvement have been described.^5^
E. Congo red stain can highlight amyloid deposits around the sweat glands and within blood vessel walls in primary systemic amyloidosis – Correct. In primary systemic amyloidosis, amyloid deposits can be observed in the dermis, subcutis, around sweat glands, and within blood vessel walls. Amyloid can be detected with multiple modalities including Congo red, crystal violet, Thioflavin T with ultraviolet fluorescence, and antiserum amyloid P antibodies.

## Conflicts of interest

None disclosed.
